# Association of Helicobacter *pylori* and Epstein-Barr virus co-infection in gastric cancer patients: a prospective analytical study

**DOI:** 10.1590/0102-672020260000016e1945

**Published:** 2026-07-10

**Authors:** Anbarasan SIVAMOORTHY, Sathasivam SURESHKUMAR, Amaranathan ANANDHI, Rahul DHODAPKAR, Palanivel CHINNAKALI, Vikram KATE

**Affiliations:** 1 Jawaharlal Institute of Postgraduate Medical Education and Research, Surgery – Pondicherry, India.; 2 Jawaharlal Institute of Postgraduate Medical Education and Research, Microbiology – Pondicherry, India.; 3 Jawaharlal Institute of Postgraduate Medical Education and Research, Preventive and Social Medicine – Pondicherry, India.

**Keywords:** Stomach neoplasms, Helicobacter pylori, Coinfection, Epstein-Barr virus infections, Neoplasias gástricas, Helicobacter pylori, Coinfecção, Infecções por vírus Epstein-Barr

## Abstract

**Background:**

Recent evidence suggests that Epstein-Barr virus (EBV) and *Helicobacter pylori* co-infection increase the prevalence of gastric cancer in the younger age group and are associated with poor prognosis. Identifying the association between these agents has important implications for the management of gastric cancer and also for defining populations at high risk of developing gastric malignancy.

**Aims:**

To determine the prevalence of *H. pylori* and EBV co-infection in patients with gastric cancer.

**Methods:**

This is a single-center prospective analytical study. A total of 182 patients were included. The study group (n=91) comprised all consecutive patients of age ≥18 years with gastric cancer. The control group (n=91) included individuals with normal endoscopy findings. Both groups were analyzed for the presence of *H. pylori* and EBV.

**Results:**

The overall prevalence of *H. pylori* infection in gastric cancer patients was 70.3%, EBV infection was 63.7%, and *H. pylori* and EBV co-infection was 51.6%. The *H. pylori* and EBV co-infection in the study and control groups was 51.6% *versus* 13.1% (p<0.001). The remaining parameters such as smoking, socioeconomic class, dietary habits, prior gastric surgery, tumor location, histological subtype, stage of the tumor, distant metastasis, and lymph node metastasis did not show any significance.

**Conclusions:**

There was a significantly higher prevalence of EBV infection and *H. pylori* and EBV co-infection in patients with gastric cancer. The prognostic and therapeutic role of co-infection requires long-term follow-up and assessment of treatment response.

## ARTICLE HIGHLIGHTS

- *Helicobacter* pylori and Epstein–Barr virus (EBV) are common in gastric cancer; and co-infection is not rare;
- Infection rates in gastric cancer are higher than in patients with normal endoscopy;
- Co-infection shows no consistent association with tumor site, Lauren subtype, or stage;
- Routine reporting of *H. pylori* and EBV status is feasible and adds useful etiologic context;
- Results support eradication-focused prevention and risk-stratified follow-up in endemic settings.

## Summary

**CENTRAL MESSAGE:**

The strongest known environmental risk factor for developing gastric cancer is prolonged colonization with *Helicobacter pylori*. More than 50% of the world’s population harbors *H. pylori* in the gastric mucosa. Epstein-Barr virus (EBV), which is associated with many hematological malignancies, is now considered to be associated with the development of gastric cancer. Studies have reported that 9% of gastric cancer cases are associated with EBV infection. The association of EBV and *H. pylori* co-infection in gastric cancer has not been extensively investigated.

**PERSPECTIVES:**

Epstein-Barr virus (EBV) infection appears to be more prevalent in gastric cancer compared to the normal population. Also, most of the gastric cancer patients have co-infection of both *Helicobacter pylori* and EBV. The importance of this observation indicates that *H. pylori* eradication alone does not help in the prevention of gastric cancer. EBV infection, which augments the damage caused by *H. pylori*, requires a specific targeted therapy. In the future, individuals with a high risk of gastric cancer may benefit from the targeted drug against the EBV-lytic cycle protein, with the aim of preventing reactivation of the virus.

## VISUAL ABSTRACT

**Figure:**
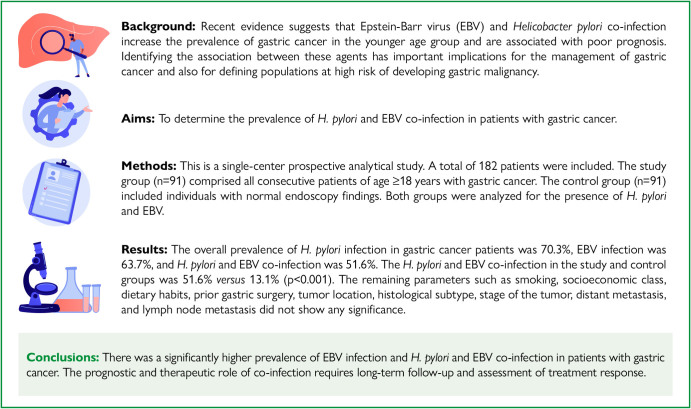

## RESUMO

**Racional:**

Evidências recentes sugerem que a coinfecção pelo vírus Epstein-Barr (EBV) e *Helicobacter pylori* aumenta a prevalência de câncer gástrico em faixas etárias jovens e está associada a pior prognóstico. Identificar a associação entre esses agentes tem importantes implicações no manejo do câncer gástrico e na definição de populações com alto risco de desenvolver malignidade gástrica.

**Objetivos:**

Determinar a prevalência de coinfecção por *H. pylori* e EBV em pacientes com câncer gástrico.

**Métodos:**

Trata-se de estudo analítico prospectivo de centro único. Ao todo, 182 participantes foram incluídos. O grupo de estudo (n=91) compreendeu todos os pacientes consecutivos com idade ≥18 anos e diagnóstico de câncer gástrico. O grupo controle (n=91) incluiu indivíduos com achados endoscópicos normais. Ambos os grupos foram analisados quanto à presença de *H. pylori* e EBV.

**Resultados:**

A prevalência de infecção por *H. pylori* nos pacientes com câncer gástrico foi de 70,3%, a infecção por EBV foi de 63,7% e a coinfecção por *H. pylori e* EBV foi de 51,6%. A coinfecção por *H. pylori* e EBV nos grupos estudo e controle foi de 51,6% *versus* 13,1% (p<0,001). Os demais parâmetros como tabagismo, classe socioeconômica, hábitos alimentares, cirurgia gástrica prévia, localização tumoral, subtipo histológico, estágio do tumor, metástases à distância e linfonodais, não mostraram significância.

**Conclusões:**

Houve prevalência significativamente maior de infecção por EBV e de coinfecção por *H. pylori* e EBV em pacientes com câncer gástrico. O papel prognóstico e terapêutico da coinfecção requer seguimento em longo prazo e avaliação da resposta ao tratamento.

## INTRODUCTION

Globally, gastric cancer is the third leading cause of cancer-related mortality, accounting for more than 700,000 deaths per year^26^. The strongest known environmental risk factor for developing gastric cancer is prolonged colonization with *Helicobacter pylori*. More than 50% of the world’s population harbors *H. pylori* in the gastric mucosa. However, only 20% of the *H. pylori* infected individuals develop gastric cancer^2^.

Epstein-Barr virus (EBV), which is associated with many hematological malignancies, is now considered to be associated with the development of gastric cancer^20^. Studies have reported that 9% of gastric cancer cases are associated with EBV infection^22^. There is a possible role of EBV and *H. pylori* as a single agent as well as in combination in the development of gastric cancer^1^.

The association of EBV and *H. pylori* co-infection in gastric cancer has not been extensively investigated. EBV infection persists in B-cells for a longer period as the virus remains in a latent stage in these cells. A 2013 analysis showed a correlation between severe gastric inflammation and increased serum EBV antibodies in the pediatric population^5^. Another study found a large number of EBV-associated gastric cancer patients who harbor both *H. pylori* and EBV infection^27^.

Recent studies suggest that EBV and *H. pylori* co-infection increases the prevalence of gastric cancer in the younger age group and is associated with poor prognosis^30^. However, only a few studies have been done on EBV and *H. pylori* co-infection in gastric cancer to date. Identifying the association between *H. pylori* and EBV has important implications for the management of gastric cancer and also for defining populations at high risk of developing gastric cancer.

The present study was carried out to determine the association of *H. pylori* and EBV co-infection in gastric cancer and its association with tumor characteristics.

## METHODS

This is a prospective, cross-sectional, analytical study conducted in the department of surgery at a tertiary care hospital from October 2017 to October 2019. It was approved by the Ethics Committee of the Institution (approval number: JIP/IEC/2018/016) and has been performed in accordance with the ethical standards laid down in an appropriate version of the Declaration of Helsinki.

The participants were recruited into the study and control groups. The study group comprised all consecutive patients of age ≥18 years with histologically proven gastric malignancy. Controls were those whose upper gastrointestinal endoscopy was normal.

Patients who had received any form of *H. pylori* eradication treatment in the past, those with severe cardiopulmonary, liver, or renal disease, recent episodes of upper gastrointestinal bleeding, previous gastric surgery, and a history of fever, maculopapular rash, and sore throat were excluded from the study. Those included were selected by convenience sampling after informed consent. All recruited individuals who fulfilled the inclusion criteria underwent upper gastrointestinal endoscopy (UGIE). Two percent lignocaine viscous or spray was used as a local anesthetic, allowing a contact time of 5 minutes. Standard endoscopic biopsy forceps were used for obtaining gastric mucosal specimens.

A total of four biopsy samples from the gastric corpus (2 samples) and the antrum (2 samples) were taken in the cases and controls. In patients with gastric cancer, the biopsy was taken from the greater curvature of the body because of better sensitivity in the detection of *H. pylori*. If the tumor involved the entire body and antrum of the stomach, the biopsy was taken from the tumor-free site^17^.

The urease test was done on two samples using a urea solution prepared and standardized in the Institute^14^. The other two gastric mucosal biopsies were fixed in 10% formalin. Giemsa staining was done in paraffin-embedded histological blocks for histological confirmation of *H. pylori*. The combination of these two tests was used as a gold standard for the diagnosis of *H. pylori* infection. If either or both the tests were positive, the patient was considered to be positive for *H. pylori* and if both the tests were negative, the patient was considered negative for *H. pylori*.

For detecting EBV infection, 5 ml of blood was taken from the gastric cancer patients and those who had normal upper gastrointestinal endoscopy. Serum was separated from the collected blood sample using a centrifuge machine and tested for EBV with the help of viral capsid antigen (VCA) and Epstein-Barr nuclear antigen (EBNA) using a specific miniVIDAS® kit (Biomerieux Diagnostics, Marcy-l’Étoile, France). A volum of 100 μl of serum was pipetted and introduced into the reagent strip and then loaded into the miniVIDAS® machine along with an appropriately controlled calibrator. Results were noted after 40 minutes of processing. Once the assay was completed, the computer analyzed the results automatically. A test value above 0.21 was considered to be positive and a value of 0.10 to 0.20 was considered to be equivocal. Those who had EBNA IgG positive and equivocal test and VCA IgG positive test had a past infection of EBV^7,9^.

A sample size of 91 in each group was calculated assuming the expected prevalence of *H. pylori* and EBV co-infection as 40% and 20% among the patients with and without gastric cancer, respectively, with alpha error of 5% (95% significance level), power of 80%, and a non-response rate of 10%, using OPENEPI® (Open Source Epidemiologic Statistics, Fleiss with continuity correction) software^3^.

### Statistical analysis

Data were entered into Microsoft Excel sheets and analyzed using Statistical Package for the Social Sciences (SPSS), version 19. Continuous variables like age were expressed as means and the difference was assessed using the *χ* ^2^ test. Categorical variables like gender, smoking, prior gastric surgery, and diet were summarized as a proportion (percentages) and the difference was analyzed using the Chi-square test. Clinicopathological features like tumor location, histological subtype, stage of gastric carcinoma, distant metastasis, and lymph node metastasis were summarized as proportion (percentages) and differences were tested using the Chi-square test. The presence of *H. pylori* and EBV co-infection in both the groups was summarized as proportion and the difference was compared using the *χ* ^2^ test. p<0.05 was considered statistically significant.

## RESULTS

A total of 182 patients were included in the study. The mean age in patients with gastric cancer and the control group was 55, standard deviation (±) 11.3 and 45±12.3 years, respectively (p=0.005). There were 70 males (78%) and 21 females (22%) in the gastric cancer group, while the control group comprised 50 males (55%) and 41 females (45%) (p<0.0009).

Table 1 shows the comparison of the prevalence of *H. pylori*, EBV, and *H. pylori* and EBV co-infection in patients with gastric cancer and controls. The prevalence of *H. pylori* infection in patients with gastric cancer was significantly higher at 70.3% when compared to that of controls at 13.1% (p<0.001). The prevalence of EBV infection in patients with gastric cancer was significantly higher at 63.7% when compared to that of controls at 23% (p<0.001). The co-infection of *H. pylori* and EBV was also higher in patients with gastric cancer (51.6%) when compared to that of controls at 13.1% (p<0.001).

**Table 1:** Prevalence of *Helicobacter pylori*, Epstein-Barr vírus, and *Helicobacter pylori* and Epstein-Barr virus co-infection in gastric cancer.

*H. pylori*	64 (70.3)	12 (13.1)	<0.00001
EBV	58 (63.7)	21 (23.0)	<0.00001
*H. pylori* and EBV co-infection	47 (51.6)	12 (13.1)	<0.00001

*χ ^2^ test. *H. pylori: Helicobacter pylori*; EBV: Epstein-Barr virus.

Table 2 shows the comparison of clinicopathological features of gastric cancer with *H. pylori* infection, EBV infection, and co-infection. It was found that the tumor characteristics such as tumor location, histological subtype, stage, distant metastasis, and lymph node metastasis were similar in patients with *H. pylori* infection, EBV infection, and *H. pylori* and EBV co-infection.

**Table 2:** Comparison of clinicopathological features with *Helicobacter pylori*, Epstein-Barr vírus, and co-infection in gastric carcinoma (n=91).

Feature	*H. pylori* positive n (%)	p-value*	EBV positive n (%)	p-value*	Co-infection positive n (%)	p-value*
Tumor location						
Cardiac (n=10)	4 (40)	0.603	4 (40)	0.436	4 (40)	0.649
Non-cardiac (n=80)	60 (75.0)		53 (66.3)		41 (51.2)	
Others (n=1)	1 (100)		1 (100)		1 (100)	
Histological subtype						
Intestinal (n=47)	34 (72.3)	0.357	28 (59.6)	0.343	22 (46.8)	0.444
Diffuse (n=41)	29 (70.7)		27 (65.9)		24 (58.5)	
Mixed (n=3)	2 (66.7)		0 (0)		1 (33.3)	
Tumor stage						
Early (n=25)	16 (64)	0.416	19 (76)	0.134	50	0.609
Locally advanced (n=66)	48 (72.7)		39 (59.1)		56	
Presence of distant metastasis (n=62)	45 (72.6)	0.492	39 (62.9)	0.809	14 (48.2)	0.660
Presence of lymph node metastasis (n=72)	54 (75.0)	0.058	45 (62.5)	0.633	39 (54.1)	0.349

* *χ* ^2^ test. *H. pylori: Helicobacter pylori*; EBV: Epstein-Barr virus.

Similarly, the risk factors such as gender, socioeconomic class, smoking, dietary habits, and prior surgery were also comparable in patients with *H. pylori* infection, EBV infection, and *H. pylori* and EBV co-infection (Table 3).

**Table 3:** Comparison of risk factors with *Helicobacter pylori*, Epstein-Barr virus, and *Helicobacter pylori*, and Epstein-Barr virus co-infection in gastric carcinoma.

Risk factor	*H. pylori* positive n (%)	p-value*	EBV positive n (%)	p-value*	Co-infection n (%)	p-value*
Gender						
Male (n=71)	52 (73.2)	0.252	47 (66.2)	0.358	39 (55.0)	0.238
Female (n=20)	12 (60.0)		11 (55.0)		8 (40.0)	
Socio-economic class						
Lower (n=36)	27 (75.0)	0.705	27 (75.0)	0.056	23 (63.9)	0.060
Upper-lower (n=22)	16 (72.7)		16 (72.7)		13 (59.1)	
Lower-middle(n=26)	16 (61.5)		12 (46.2)		8 (30.8)	
Upper-middle (n=7)	5 (71.4)		3 (42.9)		4 (57.1)	
Smoking						
Smoker (n=51)	35 (68.6)	0.688	34 (66.7)	0.511	26 (51.0)	0.886
Non-smoker (n=40)	29 (72.5)		24 (60.0)		21 (52.5)	
Dietary habits						
Vegetarian (n=5)	4/(80.0)	0.626	3/(60.0)	0.858	3/(60.0)	0.701
Mixed (n=86)	60 (69.8)		55 (63.9)		44 (51.2)	
Previous history of surgery(n=6)	5 (83.3)	0.471	4 (66.7)	0.877	3 (50.0)	0.933

* *χ* ^2^ test. *H. pylori: Helicobacter pylori*; EBV: Epstein-Barr virus.

## DISCUSSION

*H. pylori* is an important environmental risk factor for the development of gastric cancer and is considered a type 1 carcinogen based on the International Agency for Research on Cancer (IARC) classification^13^. EBV is associated with gastric cancer, and the mechanism postulated is the disruption of the genes involved in cell cycle regulation, inflammation, and the loss of tumor suppressor genes via hypermethylation^10^. The Cancer Genome Atlas classified EBV-associated gastric cancer as a separate entity due to the specific pathogenesis and tumor characteristics and also examined the differences in prognosis^31^. A better understanding of the molecular genetics and oncogenic potential of *H. pylori* and EBV is essential for appreciating the possible symbiosis between these two organisms and the resultant increase in the risk of developing gastric cancer^15^.

This study was carried out to evaluate the association of *H. pylori* and EBV co-infection with gastric cancer and found that the ocurrence of this co-infection in gastric cancer was significantly higher than that of the normal population. The frequency of *H. pylori* infection in patients with gastric cancer was high (70.3%) compared to controls (13.1%), corroborating a study done by Kate et al.^14^. A similar high prevalence of *H. pylori* has also been noted in other works in the literature, indicating that there is an association between H. pylori infection and gastric cancer^24,29^.

*H. pylori* is known to cause persistent chronic inflammation of the gastric antrum and lead to atrophic gastritis, which in turn causes achlorhydria and alkaline pH. This micro-environment increases the proliferation of *H. pylori* and the risk of gastric cancer^17^.

The prevalence of EBV infection in patients with gastric cancer, in the current study, was found to be significantly higher than in the controls (63.7 *versus* 23%; p<0.001). Most of the patients with gastric cancer had high serum titers of EBNA and VCA IgG antibodies, which indicates that all patients had a past EBV infection in childhood. However, a Peruvian research on patients with chronic gastritis and gastric cancer, found a much lower prevalence of EBV infection compared to our study (19.2%). The lower prevalence in their analysis may be due to the use of polymerase chain reaction (PCR) for EBV detection and regional variation in EBV rates^6^. On the other hand, Nogueira et al. found a high prevalence of EBV infection in patients with gastric cancer, using the PCR technique for the diagnosis of EBV infection^24^. However, when *in situ* hybridization techniques were used for the diagnosis of EBV infection, 11% of the patients showed positivity for EBV infection, indicating a lower sensitivity of *in situ* hybridization techniques than PCR^25^. A systematic review analyzing 47 studies showed that seropositivity of EBV-specific antibodies was significantly higher among gastric carcinoma patients^7^.

The prevalence of *H. pylori* and EBV co-infection in gastric cancer, in our study, is higher compared to the normal population and the difference is statistically significant (p<0.001). The *H. pylori* and EBV co-infection was also found to be significantly higher within the gastric cancer population (p<0.001). A study on the South American population showed a similar prevalence of co-infection in gastric cancer compared to patients with chronic gastritis. The authors found that the reactivation of EBV antigen (by measuring reactivation antibody) is necessary for gastric inflammation and suggested that the presence of *H. pylori* alone does not contribute to the inflammation. It is clear from the above evidence that EBV reactivation is required for inducing severe inflammation in gastric mucosa along with the *H. pylori*, proving the complementary effect of *H. pylori* and EBV co-infection in the carcinogenesis of gastric cancer^5^.

Recently, a study done in India showed that co-infection increases the incidence of gastric carcinoma and is associated with onset of gastric cancer at a younger age compared with individual infection^30^.

This possible association between *H. pylori* and EBV co-infection is due to the increased expression of IL-17 (interleukin-17) which induces a sustained chronic inflammatory state leading to gastric mucosal damage. Pandey et al. studied extensively the molecular pathogenesis of *H. pylori* and EBV and first proposed the possible complementary action of *H. pylori* and EBV co-infection in the oncogenesis of gastric cancer^25^. They established that *in vitro*, *H. pylori* associated CagA (cytotoxin-associated gene A) induces phospholipase C gamma (PLC) activation in EBV latent B lymphocytes, which in turn causes EBV reactivation and induces greater infiltration of B cells loaded with viral particles into the gastric mucosa. Thereby, dual infection with *H. pylori* and EBV activates the ß-catenin/TCF-4 (Transcription Factor-4) pathway, which is the transforming factor in gastric cells and leads to an increased risk of gastric cancer^25^.

Only a few studies have evaluated the association of *H. pylori* and EBV co-infection in gastric carcinoma, with inconsistent results on the association between the two pathogens in tumorigenesis. Camargo et al. also suggested that *H. pylori* enhances the latency of EBV in gastric mucosa by dampening the host oxidative stress response^4^.

Thus, *H. pylori* infection enhances the proliferation of EBV infection in gastric epithelium through the interaction of the cell cycle, apoptosis, and DNA damage repair pathways, ultimately leading to uncontrolled proliferation of EBV-infected cells and the development of gastric cancer. Also, EBV infection enhances the oncogenic potential of phosphorylated CagA and indicates the role of the virus in the pathogenesis of gastric cancer^27^. A few studies also identified that the *H. pylori* infection causes EBV reactivation in the gastric epithelial cells^21,23^.

The concept of complementary action was also established by Pandey et al.^25^ who investigated the EBV-driven epigenetic modifications in gastric cancer and found that the presence of CagA secretory antigen of H. *pylori* enhances the EBV-driven epigenetic change. A study done in Northern India showed a similar higher prevalence of *H. pylori* and EBV in gastric carcinoma (54%) and the EBV DNA copy number was significantly higher in *H. pylori* positive gastric cancer^30^.

Inconsistent correlations have also been shown by various studies on the association of *H. pylori* and EBV concerning the histological type. Naseem et al.^23^ found that the diffuse type frequently occurs in EBV-associated gastric cancer. Analysis of published literature shows that both intestinal type and diffuse type histology can be found in EBV-associated gastric cancer. A recent meta-analysis and a few other studies also found EBV association in both intestinal and diffuse subtypes^8,16,18,22^. This is because *H. pylori* stimulates the release of pro-inflammatory cytokines, particularly IL-8 (interleukin-8) and IL-1 (interleukin-1), from the gastric epithelial cells leading to severe mucosal damage. The pooled meta-analysis of 12 studies done by *H.* and Cancer Collaborative Group also found that *H. pylori was* associated with intestinal and diffuse type in a large number of research^11^. The prevalence of *H. pylori* and EBV co-infection, in the present study, showed no significant difference between intestinal and diffuse types (p>0.05). Many studies failed to show a significant association of co-infection with a particular histological subtype^12,24^.

Most of the gastric cancers were located in the non-cardiac region and half of them had co-infection in the present study. The review of literature shows that *H. pylori-*associated gastric cancer is predominant in the antral region. This is possibly due to the *H. pylori* virulence factor CagA gene augmenting the inflammation in the antrum. Huang et al. also showed a similar association of CagA-positive strains of *H. pylori* from the antral region of gastric cancer^28^. In North America, EBV-associated gastric cancer occurs frequently in the cardiac region^30^. The tumor location was not found to be a significant factor in the presence of co-infection by univariate analysis in our study.

Most cases of gastric cancer was locally advanced and half of them were positive for *H. pylori* and EBV co-infection. No significant difference was observed between early- and advanced-stage tumors among co-infected patients. Distant metastases were uncommon, whereas lymph node involvement was more frequent.

Even though the literature review suggests that EBV-associated tumors have a good prognosis, in our analysis there was no significant difference found between the co-infection and stage of the disease, distant and lymph node metastasis. Few reports showed better median survival and disease-free survival (DFS) among EBV-associated gastric cancer^4,32^. An investigation conducted on the Asian population showed a better overall survival of EBV-associated gastric cancer among the Asian population^19^. In our study, many patients with lymph node metastasis had co-infection; however, the difference was not significant. Since the present study did not follow the patients to assess the treatment response, overall survival, and disease-free survival, the prognosis implicated by co-infection could not be established.

The merits of our study include a prospective analytical design conducted in a tertiary care hospital with a high number of gastric cancer patients. The Enzyme-Linked Immunosorbent Assay (ELISA) was employed for EBV detection, which has been shown to have lower specificity in spite of higher sensitivity^29^. The PCR could have been a better method for the detection of *H. pylori* and EBV, which has greater sensitivity and specificity; however, the PCR technique was not used due to logistic constraints.

## CONCLUSIONS

From the above observations, EBV infection appears to be more prevalent in gastric cancer compared to the normal population. Also, most of the gastric cancer patients had co-infection with both *H. pylori* and EBV. The importance of this observation is that *H. pylori* eradication alone does not help in the prevention of gastric cancer. EBV infection, which augments the damage caused by *H. pylori*, requires specific target therapy. In the future, individuals with a high risk of gastric cancer may benefit from targeted drugs against the EBV-lytic-cycle proteins, with the aim of preventing viral reactivation.

## Data Availability

The datasets generated and/or analyzed during the current study are available from the corresponding author upon reasonable request.
